# Urinary Excretion of Kidney Aquaporins as Possible Diagnostic Biomarker of Diabetic Nephropathy

**DOI:** 10.1155/2017/4360357

**Published:** 2017-01-26

**Authors:** Luigi Rossi, Maria Celeste Nicoletti, Monica Carmosino, Lisa Mastrofrancesco, Antonella Di Franco, Francesca Indrio, Rossella Lella, Luigi Laviola, Francesco Giorgino, Maria Svelto, Loreto Gesualdo, Giuseppe Procino

**Affiliations:** ^1^DETO, University of Bari, Bari, Italy; ^2^Department of Biosciences, Biotechnologies and Biopharmaceutics, University of Bari, Bari, Italy

## Abstract

Diabetic nephropathy (DN) is a microangiopathic complication of diabetes mellitus (DM) affecting one-third of diabetic patients. The large variability in the clinical presentation of renal involvement in patients with DM makes kidney biopsy a prerequisite for a correct diagnosis. However, renal biopsy is an invasive procedure associated with risk of major complications. Numerous studies aimed to identify a noninvasive biomarker of DN but, so far, none of these is considered to be sufficiently specific and sensitive. Water channel aquaporins (AQPs), expressed at the plasma membrane of epithelial tubular cells, are often dysregulated during DN. In this work, we analyzed the urine excretion of AQP5 and AQP2 (uAQP5 and uAQP2),* via* exosomes, in 35 diabetic patients: 12 normoalbuminuric with normal renal function (DM), 11 with proteinuric nondiabetic nephropathy (NDN), and 12 with histological diagnosis and classification of DN. ELISA and WB analysis independently showed that uAQP5 was significantly increased in DN patients. Interestingly, linear regression analysis showed a positive correlation between uAQP5 and the histological class of DN. The same analysis, focusing on uAQP2, showed comparable results. Taken together, these data suggest a possible use of AQP5 and AQP2 as novel noninvasive biomarkers to help in classifying the clinical stage of DN.

## 1. Introduction

Diabetes mellitus (DM) affects over 350 million people worldwide and its prevalence and incidence are growing, thus suggesting the concept of a* diabetic pandemic*. Diabetic patients have twice the risk of dying of cardiovascular complication compared to nondiabetic subjects of the same age [1, 2].

Diabetic nephropathy (DN) is a slow, progressive loss of kidney function caused by long-standing DM, both type I and type II. DN affects only one-third of diabetic patients, suggesting the concomitant involvement of environmental and genetic factors contributing to the initiation and progression of diabetic kidney disease [3, 4].

It is very important to emphasize the concept that DN is only one of the possible forms of kidney damage occurring in diabetic patients. In fact, renal damage in these patients may be due to other causes that can coexist with the diabetic damage or even represent the only pathogenic factor in over 40% of diabetic patients [5]. DN is classified into four hierarchical glomerular lesions with a separate evaluation for degrees of interstitial and vascular involvement [6, 7].

In renal diseases, except those due to congenital abnormalities and obstructions, only kidney biopsy allows an etiologic diagnosis. This is clearly an invasive method and, for this reason, its use is limited to selected cases. Currently, microalbuminuria is the only, early, noninvasive marker of endothelial dysfunction in kidney. Microalbuminuria is defined as small quantities of albumin in the urine, ranging from 30 to 300 mg/day. This value is very well accepted in both type I and type II DM not only as diagnostic biomarker but also as prognostic and pathogenic factor [8–10].

However, microalbuminuria has important limits: first of all it does not allow an etiologic diagnosis of DN, because it is just a marker of endothelial damage; secondly, it does not relate type and amount of kidney damage; finally, it is not an essential condition associated with DN: in fact, it is known that some diabetic patients worsen to renal failure without showing albuminuria [11, 12].

Other proteins than albumin have been proposed as diagnostic marker of DN: *β*2 microglobulin (*β*2MG) and ubiquitin [13]. Nevertheless, these proteins have also limitations: *β*2MG is a marker of tubular damage, and until now there are no studies comparing urinary *β*2MG excretion in diabetic versus nondiabetic nephropathy patients. Also ubiquitin relevance as diagnostic marker is limited by the lack of correlation between its urinary levels and histologic classification of DN.

The majority of the studies, undertaken to identify novel urinary biomarkers, focused on the analysis of soluble proteins in the urine; more recently, the possibility to search for novel biomarkers associated with nanoscale vesicles known as exosomes, released into the urinary space, is emerging. This approach opens new perspective for better understanding the molecular mechanisms leading to renal damage and to discover new biomarkers [14, 15].

Novel ideal biomarkers of DN should be characterized by sensitivity, specificity, early appearance, and low invasiveness. The water channels aquaporins (AQPs) may have a relevant role in the establishment and maintenance of DN because of their role in the regulation of fluid balance in the kidney and since polyuria is a very early clinical sign of diabetes.

Among the 13 isoforms cloned in humans, at least 9 are expressed in the kidney: AQP1-8 and AQP11 [16, 17].

AQP1 [18] is highly expressed in the glomerular capillaries at the plasma membrane of proximal tubule epithelial cells [19]. AQP2 [20] is expressed at the apical plasma membrane of principal cells (PC) of collecting duct (CD) during antidiuretic hormone (ADH) stimulation [17]. We previously showed that, under physiological conditions, AQP5 is expressed in the intercalated *β*-cells of the CD [21].

Early reports showed that renal AQPs might be dysregulated in diabetic patients and in DN [22–24]. Wu et al. showed that AQP5 is upregulated in kidney biopsies from DN patients [25]. On the basis of this evidence, in this work, we analyzed the urinary excretion of AQP1, AQP2, and AQP5 in three groups of patients: 12 diabetic with no sign of nephropathy (DM), 12 diabetic with histologic diagnosis of diabetic nephropathy (DN), and 11 diabetic with nondiabetic nephropathy (NDN).

In this pilot investigation, we found that urine excretion of AQP2 and AQP5, but not AQP1, was dramatically higher in DN patients compared to DM and NDN patients and positively correlated with the progression of the DN, according to the histologic classification.

Taken together, these data support a possible application of urinary excreted AQP2 and AQP5 as ideal biomarkers for the diagnosis of DN.

## 2. Materials and Methods

### 2.1. Study Participants

In this study, we enrolled 35 patients to evaluate urinary excretion of AQP2, AQP5, AQP1, and NKCC2. All patients were affected by type 2 diabetes mellitus. The main demographic, clinical, and laboratory features are summarized in Table 1.

Seven healthy (nondiabetic) volunteers were also recruited as controls. They were not hospitalized and provided urine samples.

The following four groups entered the study:

1. 7 control healthy patients with normal glycemia, GFR, blood pressure, and albuminuria (CTR)
2. 12 diabetic patients with no apparent sign of nephropathy, normal GFR, and normoalbuminuria (DM)
3. 12 diabetic patients with biopsy-proven diabetic nephropathy (DN), classified according to the criteria proposed by Tervaert et al. [7] and reported in Table 2
4. 11 diabetic patients with nondiabetic nephropathy (NDN) described in Table 2.

### 2.2. Ethics Statements

The design of the study was observational, since no additional intervention was planned apart from routine medical care. Samples were collected after the patients gave their written informed consent and the study was reviewed and approved by the Institutional Review Board of the Polyclinic Hospital, University of Bari Medical School, Bari (Italy) (study number ST 3325, prot. 823 approved on 20 July 2009), and it was carried out in accordance with the Helsinki Declaration of 1975 (as revised in 2000). No individual patient data are reported in this article.

### 2.3. Urine and Tissue Collection

All samples from all patients and healthy volunteers enrolled in this study were collected at the Nephrology, Dialysis and Transplant Department of the University Hospital “Policlinico di Bari,” Bari (Italy). First morning urine samples were supplemented with Protease Inhibitor Cocktail Tablets (Roche Diagnostics GmbH), centrifuged at 3,000 rpm for 10 minutes at 4°C to remove cellular debris, and stored at −80°C. Urine creatinine was quantified by the Jaffe reaction.

Patients diagnosed with diabetic nephropathy and nondiabetic nephropathy underwent kidney biopsy. Control human kidneys samples were obtained from the normal-appearing renal cortex harvested from a nondiabetic patient diagnosed with renal carcinoma and undergoing a nephrectomy with approval of the local ethics committee. At time of biopsy, all patients were clinically stable regarding water balance and blood pressure. All patients gave signed consent for the use of their tissue for research purposes at the time of biopsy or radical nephrectomy.

### 2.4. Antibodies

Rabbit affinity purified polyclonal antibody against amino acids 251–265 of rat AQP5 (cat.# AQP5-005) was from Alomone Labs (http://www.alomone.com). The antibody is designed to cross-react also with human AQP5. We tested the absence of cross-reaction of the anti-AQP5 antibody with AQP2 (data not shown). Rabbit polyclonal antibody against human AQP2 was previously described [26]. Rabbit anti-AQP1 antibody (cat.# SC-20810) and rabbit anti-flotillin-1 antibody (cat.# sc-25506) were from Santa Cruz Biotechnology (http://www.scbt.it). Rabbit anti-NKCC2 polyclonal antibody (cat.# AB3562P) and monoclonal anti-Na^+^/K^+^-ATPase (cat.# 05-369) were from Millipore (http://www.merckmillipore.com). Anti-rabbit IgG (whole molecule)-peroxidase antibody (cat.# A0545) was from Sigma-Aldrich (http://www.sigmaaldrich.com).

### 2.5. Immunofluorescence

Human kidney samples were fixed in 4% paraformaldehyde in PBS at 4°C, embedded in paraffin wax, and cut into 5 *μ*m sections with a microtome. Antigen retrieval was performed by boiling sections in citrate buffer (10 mM sodium citrate, 0,05% Tween 20, pH 6) for 30 minutes. Nonspecific binding sites were blocked with 1% BSA in PBS for 30 min. Sections were then incubated with rabbit anti-AQP2 antibody (1 : 1000) followed by incubation with Alexa Fluor-conjugated secondary antibody (http://www.lifetechnologies.com). Confocal images were obtained with a confocal laser-scanning microscope (Leica TSC-SP2, Mannheim, Germany).

### 2.6. Purification of Urine Exosomes and Western Blotting Analysis

Isolation of exosomes from urine was performed using a two-step differential centrifugation method [27]. Briefly, an equal volume of urine samples (1 mL) was centrifuged at 17,000 ×g for 10 min at 4°C to remove urinary sediment and supernatants were ultracentrifuged at 200,000 ×g for 1 hour at 4°C. The resulting exosome-enriched pellets were resuspended in 60 *μ*L of Laemmli's buffer and denatured for 10 min at 60°C.

Ten microliters of each sample was resolved on 12% SDS-PAGE and electroblotted onto Immobilon-P PVDF membrane (http://www.merckmillipore.com). After blocking with 3% BSA in Tris buffer saline/Tween 20 (TBS-T), blots were incubated overnight at 4°C with the following primary antibodies: anti-flotillin-1 (1.200), anti-Na^+^/K^+^-ATPase (1 : 5000), anti-AQP5 (1 : 1000), anti-AQP2 (2 *μ*g/mL), anti-AQP1 (1 : 500), and anti-NKCC2 (1 : 1000). Membranes were washed and incubated with the anti-rabbit IgG peroxidase antibody (1 : 10000). Reactive proteins were revealed with SuperSignal™ West Pico Chemiluminescent Substrate (https://www.thermofisher.com) and chemiluminescence was detected with ChemiDoc XRS (Bio-Rad) equipped with Image Lab™ software for image acquisition. Densitometry analysis was performed with ImageJ software (NIH, Bethesda, MD).

### 2.7. ELISA Test for uAQP5 and uAQP2

Quantitation of urinary AQP5 (uAQP5) and AQP2 (uAQP2) was performed using a standard ELISA protocol originally established by Umenishi et al., [28] with some modifications [29]. Synthetic peptides reproducing the last 15 amino acids of the C-terminal region of the rat AQP5 and human AQP2 were used as internal standard for AQP5 and AQP2 assay, respectively. Urinary AQ5 and AQP2 excretion was normalized to urinary creatinine and expressed as fmol/mg urine creatinine.

### 2.8. Statistical Analysis

Data were expressed as mean ± standard error (SE) of the mean. Statistical analysis was performed using a nonparametric Kruskal-Wallis one-way ANOVA test, linear regression test, and Spearman's rank-order correlation. *P* values < 0.05 were considered statistically significant. For statistical analysis, GraphPad Prism 5 software was used. Data were obtained from at least three independent experiments for each experimental condition.

## 3. Results

### 3.1. Urinary AQP5 Excretion

**Figure 1:**
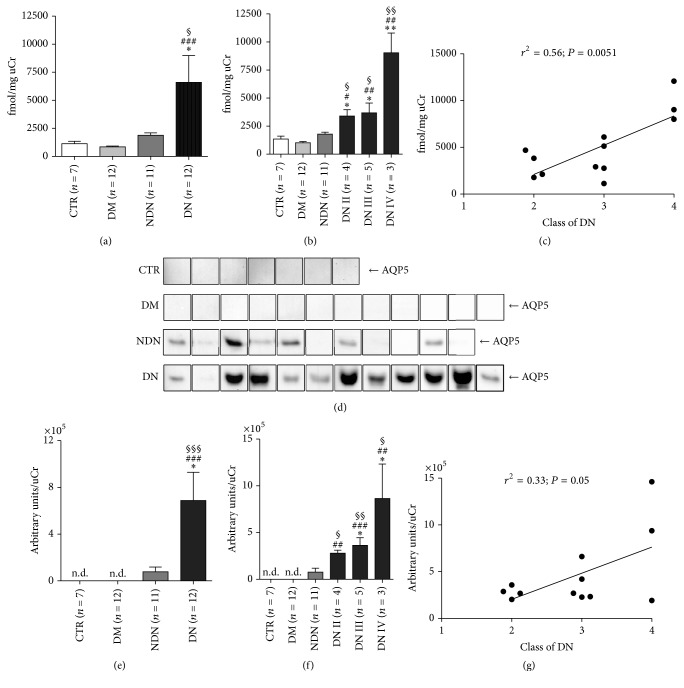
The urinary excretion of AQP5 dramatically increases in DN patients and positively correlates with the clinical severity of DN. (a) Urinary AQP5 excretion was measured by ELISA in urine samples of healthy subjects (CTR, *n* = 7), patients with DM (*n* = 12), patients with NDN (*n* = 11), and patients with DN (*n* = 12) and expressed as fmol/mg urine creatinine. All data are reported as mean ± SEM. ^§^*P* < 0.05 versus CTR, ^###^*P* < 0.001 versus DM, and ^*∗*^*P* < 0.05 versus NDN, obtained by Kruskal-Wallis one-way ANOVA test. (b) DN patients were grouped according to the histological changes evaluated by kidney biopsy: DN stage II (*n* = 4), DN stage III (*n* = 5), and DN stage IV (*n* = 3) and uAQP5 at each stage compared to uAQP5 in CTR and DM and NDN patients. All data are reported as mean ± SEM. ^§§^*P* < 0.01 and ^§^*P* < 0.05 versus CTR, ^##^*P* < 0.01 and ^#^*P* < 0.05 versus DM, and ^*∗∗*^*P* < 0.01 and ^*∗*^*P* < 0.05 versus NDN, obtained by Kruskal-Wallis one-way ANOVA test. (c) Linear regression analysis of uAQP5 abundance, as measured by ELISA, with the class of DN (*r*^2^ = 0.56; *P* = 0.0051). (d) Exosomes were isolated from urine of healthy subjects (CTR, *n* = 7), patients with DM (*n* = 12), patients with NDN (*n* = 11), and patients with DN (*n* = 12) using the two-step differential centrifugation method. Total exosome proteins were resolved on 12% SDS-PAGE and analyzed by Western blotting for AQP5 abundance. (e) Densitometry analysis of the uAQP5 band intensities was normalized for uCr and reported as means ± SEM. ^§§§^*P* < 0.001 versus CTR, ^###^*P* < 0.001 versus DM, and ^*∗*^*P* < 0.05 versus NDN, obtained by Kruskal-Wallis one-way ANOVA test. n.d.: nondetectable. (f) DN patients were grouped according to the histological changes evaluated by kidney biopsy: DN stage II (*n* = 4), DN stage III (*n* = 5), and DN stage IV (*n* = 3) and densitometry analysis of uAQP5 at each stage compared to uAQP5 in CTR and DM and NDN patients. All data are reported as mean ± SEM. ^§§^*P* < 0.01 and ^§^*P* < 0.05 versus CTR, ^##^*P* < 0.01 and ^###^*P* < 0.001 versus DM, and ^*∗*^*P* < 0.05 versus NDN, obtained by Kruskal-Wallis one-way ANOVA test. (g) Linear regression analysis of uAQP5 abundance, as semiquantified by Western blotting, with the class of DN (*r*^2^ = 0.33; *P* = 0.05).

An ELISA assay was designed and carried out to quantify uAQP5 excretion between the four groups of patients previously described. Results are reported in Figure 1(a) and expressed as fmol AQP5/mg uCr.

uAQP5-to-creatinine* ratio* was the highest in patients with diabetic nephropathy (DN) compared to patients with nondiabetic nephropathy (NDN) and diabetic subjects (DM) and healthy nondiabetic volunteers (CTR). No statistically significant difference in uAQP5 excretion was observed between CTR and DM and NDN groups.

We next investigated whether the increase in uAQP5 excretion was related with the progression of DN. To this end, we performed a post hoc subanalysis of uAQP5 excretion after grouping all DN patients according to stages II, III, and IV of DN, performed as described in Materials and Methods.

As shown in Figure 1(b), uAQP5 excretion progressively increased in parallel with the histological class of DN. uAQP5 was significantly higher in DN patients at stage II compared to CTR and DM and NDN groups. uAQP5 did not further increase in DN patients at stage III but almost doubled in DN patients at stage IV. Linear regression analysis (Figure 1(c)) showed a strong, positive association between uAQP5 and the progression of DN (*r*^2^ = 0.56; *P* = 0.0051).

Exosome-associated proteins were isolated from equal volumes (1 mL) of urine from all patients, separated by standard SDS-PAGE and analyzed by Western blotting. The results showed the presence of the exosome marker flotillin-1 [30] and the absence of the basolateral membrane marker Na^+^/K^+^-ATPase (see supplemental Figure 1 in Supplementary Material available online at https://doi.org/10.1155/2017/4360357). The Western blotting analysis also confirmed the marked increase in uAQP5 in patients with DN.

As reported in Figure 1(d), a strong band of approximately 27 kDa for AQP5 was detected in almost all urine exosomes isolated from the DN group, while a faint band was immunodetected only in six out of the eleven NDN patients. Exosomes isolated from the CTR and DM groups showed no detectable signal for AQP5. The densitometry analysis of uAQP5 bands, normalized to uCr (Figure 1(e)), indicated that uAQP5 was dramatically increased in the DN subjects when compared to CTR and DM and NDN groups.

When DN patients were grouped according to the stage of DN (Figure 1(f)), the analysis showed a tendency to a progressive increase of uAQP5 excretion with the progression of the DN, although linear regression analysis did not show a statistical significant correlation (Figure 1(g); *r*^2^ = 0.33; *P* = 0.05). Spearman's rank-order correlation between uAQP5 and uACR or glomerular filtration rate (GFR), calculated as CKD-EPI, is reported in supplemental Figure 2. In both NDN and DN patients, uAQP5 did not correlate with uACR (supplemental Figure 2, C, E). Only DN patients showed a significant, negative correlation between uAQP5 and CKD-EPI (supplemental Figure 2F).

### 3.2. Urinary AQP2 Excretion

We next evaluated whether also uAQP2 was altered in DN patients using the ELISA test as described above.

**Figure 2:**
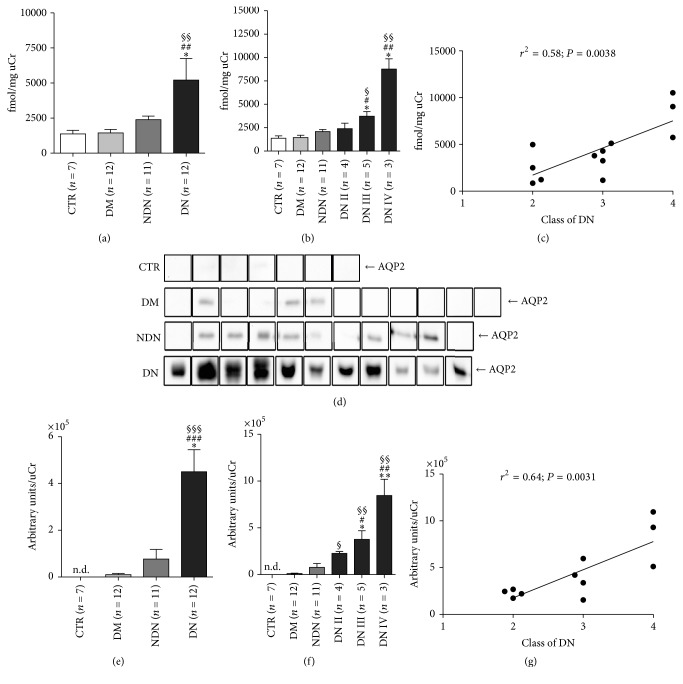
The urinary excretion of AQP2 dramatically increases in DN patients and positively correlates with the clinical severity of DN. (a) Urinary AQP2 excretion was measured by ELISA in urine samples of healthy subjects (CTR, *n* = 7), patients with DM (*n* = 12), patients with NDN (*n* = 11), and patients with DN (*n* = 12) and expressed as fmol/mg urine creatinine. All data are reported as mean ± SEM. ^§§^*P* < 0.01 versus CTR, ^##^*P* < 0.01 versus DM, and ^*∗*^*P* < 0.05 versus NDN, obtained by Kruskal-Wallis one-way ANOVA test. (b) DN patients were grouped according to the histological changes evaluated by kidney biopsy: DN stage II (*n* = 4), DN stage III (*n* = 5), and DN stage IV (*n* = 3) and uAQP2 at each stage compared to uAQP2 in DM and NDN patients. All data are reported as mean ± SEM. ^§§^*P* < 0.01 and ^§^*P* < 0.05 versus CTR, ^##^*P* < 0.01 and ^#^*P* < 0.05 versus DM, and ^*∗*^*P* < 0.05 versus NDN, obtained by Kruskal-Wallis one-way ANOVA test. (c) Linear regression analysis of uAQP2 abundance, as measured by ELISA, with the class of DN (*r*^2^ = 0.58; *P* = 0.0038). (d) Exosomes were isolated from urine of healthy subjects (CTR, *n* = 7), patients with DM (*n* = 12), patients with NDN (*n* = 11), and patients with DN (*n* = 11) using the two-step differential centrifugation method. Total exosome proteins were resolved on 12% SDS-PAGE and analyzed by Western blotting for AQP2 abundance. (e) Densitometry analysis of the uAQP2 band intensities was normalized for uCr and reported as means ± SEM. ^§§§^*P* < 0.001 versus CTR, ^###^*P* < 0.001 versus DM, and ^*∗*^*P* < 0.05 versus NDN, obtained by Kruskal-Wallis one-way ANOVA test. (f) DN patients were grouped according to the histological changes evaluated by kidney biopsy: DN stage II (*n* = 4), DN stage III (*n* = 4), and DN stage IV (*n* = 3) and densitometry analysis of uAQP2 at each stage compared to uAQP2 in CTR and DM and NDN patients. All data are reported as mean ± SEM. ^§§^*P* < 0.01 and ^§^*P* < 0.05 versus CTR, ^##^*P* < 001 and ^#^*P* < 0.05 versus DM, and ^*∗∗*^*P* < 0.01 and ^*∗*^*P* < 0.05 versus NDN, obtained by Kruskal-Wallis one-way ANOVA test. (g) Linear regression analysis of uAQP2 abundance, as semiquantified by Western blotting, with the class of DN (*r*^2^ = 0.64; *P* = 0.0031).

Similar to uAQP5, uAQP2-to-creatinine* ratio* was maximal in patients with ND compared to both CTR and DM and NDN groups (Figure 2(a)) and there was no statistically significant difference between CTR and DM and NDN patients.

A further analysis, performed after grouping DN patients according to the histological classification of DN, indicated that the most severe stages (III and IV) were characterized by a significantly higher uAQP2 excretion compared to CTR and DM and NDN subjects (Figure 2(b)). In contrast, uAQP2 in DN subjects at stage II was comparable to that of CTR and DM and NDN groups. Linear regression analysis showed a positive relationship between uAQP2 and the progression of DN (Figure 2(c); *r*^2^ = 0.58; *P* = 0.0038).

Semiquantitative Western blotting analysis of the uAQP2 abundance in urinary exosomes gave results in line with those obtained by ELISA. In fact, a protein band of approximately 29 kDa, corresponding to AQP2, was significantly more abundant in patients with DN compared to the three reference groups (Figures 2(d) and 2(e)). The post hoc statistical analysis performed on DN patients showed that uAQP2 progressively increased with the class of DN (Figure 2(f)). Compared to CTR subjects, all three classes of DN patients showed a progressive increase of uAQP2. Compared to DM and NDN patients, only DN patients assigned to classes III and IV showed higher uAQP2 excretion. Linear regression analysis confirmed that uAQP2 abundance positively correlated with the severity of DN (Figure 1(g); *r*^2^ = 0.64; *P* = 0.0031). Spearman's rank-order correlation between uAQP2 and uACR or glomerular filtration rate (GFR), calculated as CKD-EPI, is reported in supplemental Figure 3. In both NDN and DN patients, uAQP2 did not correlate with uACR (supplemental Figure 3, C, E). Only DN patients showed a significant, negative correlation between uAQP2 and CKD-EPI (supplemental Figure 3F).

Importantly, both uAQP5 and uAQP2 were correlated neither with blood pressure nor with plasma cholesterol (total, HDL, and LDL) nor with antihypertensive or antidiabetic drugs, including statins.

### 3.3. AQP2 Abundance and Subcellular Localization in Kidney Biopsies

**Figure 3:**
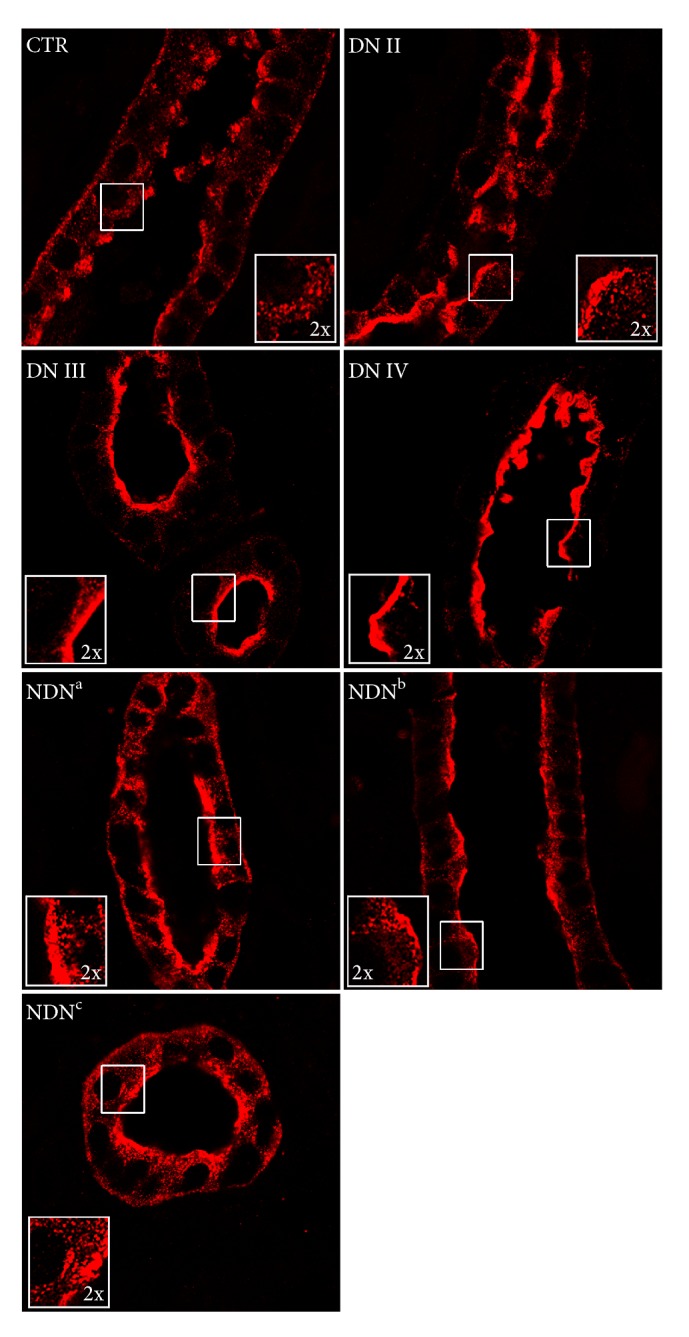
AQP2 plasma membrane localization increases in DN patients and with the clinical severity of DN. Human kidney biopsies from control kidney (CTR), DN, and NDN patients were subjected to immunofluorescence analysis of AQP2 subcellular localization. AQP2 immunostaining, shown in red, was visualized in the *xy* apical confocal plan. Confocal pictures are representative of AQP2 staining in the cortical collecting duct of normal kidney (CTR), kidneys of patients with class II (DN II), class III (DN III), and class IV (DN IV) DN, and kidneys of NDN patients. NDN^a^ is a patient with membranous nephropathy and angiosclerosis, NDN^b^ is a patient with chronic interstitial nephritis, and NDN^c^ is a patient with endocapillary/extracapillary glomerulonephritis. Similar results were obtained in three patients per each group.

AQP2 was also immunolocalized in sections of renal cortex biopsies from DN and NDN patients. As control, we used normal kidney tissue obtained as explained in Materials and Methods.  Figure 3 shows representative confocal pictures of AQP2 staining taken in the cortical collecting ducts of normal kidney, kidneys of patients with classes II, III, and IV DN, and kidneys of NDN patients. In control tissue, AQP2 was mainly localized in intracellular storage vesicles accumulated at the subapical region (magnified ×2 in the insets). In contrast, in tissues from all DN patients, AQP2 staining was brighter and progressively accumulated at the luminal plasma membrane in parallel with the histologic classification of DN (see magnified ×2 insets). AQP2 staining in different forms of NDN showed a predominant subapical localization similar to that observed in control samples.

### 3.4. Urinary AQP1 and NKCC2 Excretion

We next analyzed the urine excretion of AQP1 and NKCC2 in the three groups of patients.

**Figure 4:**
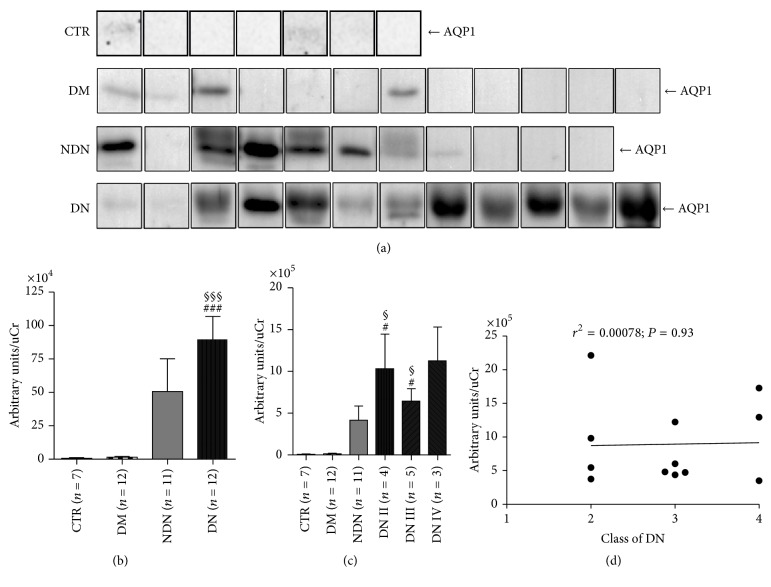
The urinary excretion of AQP1 increases in both DN and NDN patients and does not correlate with the clinical severity of DN. (a) Exosomes were isolated from urine of healthy subjects (CTR, *n* = 7), patients with DM (*n* = 12), patients with NDN (*n* = 11), and patients with DN (*n* = 12) using the two-step differential centrifugation method. Total exosome proteins were resolved on 12% SDS-PAGE and analyzed by Western blotting for AQP1 abundance. (b) Densitometry analysis of the uAQP1 band intensities was normalized for uCr and reported as means ± SEM. ^§§§^*P* < 0.001 versus CTR and ^###^*P* < 0.001 versus DM, obtained by Kruskal-Wallis one-way ANOVA test. (c) DN patients were grouped according to the histological changes evaluated by kidney biopsy: DN stage II (*n* = 4), DN stage III (*n* = 5), and DN stage IV (*n* = 3) and densitometry analysis of uAQP1 at each stage compared to uAQP1 in CTR and DM and NDN patients. All data are reported as mean ± SEM. ^§^*P* < 0.05 versus CTR and ^#^*P* < 0.05 versus DM, obtained by Kruskal-Wallis one-way ANOVA test. (d) Linear regression analysis of uAQP1 abundance, as semiquantified by Western blotting, with the class of DN (*r*^2^ = 0.00078; *P* = 0.93).

**Figure 5:**
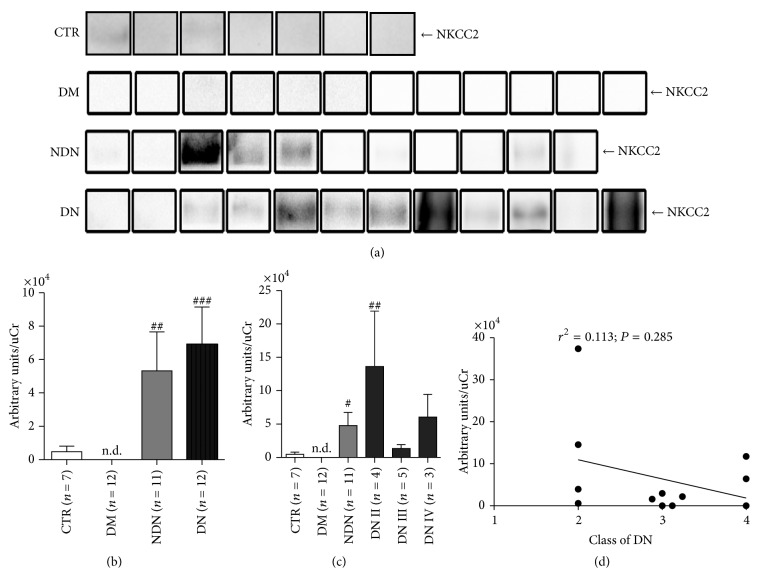
The urinary excretion of NKCC2 increases in both DN and NDN patients and does not correlate with the clinical severity of DN. (a) Exosomes were isolated from urine of healthy subjects (CTR, *n* = 7), patients with DM (*n* = 12), patients with NDN (*n* = 11), and patients with DN (*n* = 12) using the two-step differential centrifugation method. Total exosome proteins were resolved on 10% SDS-PAGE and analyzed by Western blotting for NKCC2 abundance. (b) Densitometry analysis of the uNKCC2 band intensities was normalized for uCr and reported as means ± SEM. ^###^*P* < 0.001 and ^##^*P* < 0.01 versus DM, obtained by Kruskal-Wallis one-way ANOVA test. n.d.: nondetectable. (c) DN patients were grouped according to the histological changes evaluated by kidney biopsy: DN stage II (*n* = 4), DN stage III (*n* = 5), and DN stage IV (*n* = 3) and densitometry analysis of uNKCC2 at each stage compared to uNKCC2 in CTR and DM and NDN patients. All data are reported as mean ± SEM. ^##^*P* < 0.01 and ^#^*P* < 0.05 versus DM, obtained by Kruskal-Wallis one-way ANOVA test. (d) Linear regression analysis of uNKCC2 abundance, as semiquantified by Western blotting, with the class of DN (*r*^2^ = 0.113; *P* = 0.285).

The amounts of uAQP1 and uNKCC2, excreted through the exosomes pathway, were analyzed by Western blotting (Figures 4(a)–5(a)).

As indicated by the densitometry analysis (Figure 4(b)), uAQP1, normalized for the uCr, was significantly increased in DN compared to CTR and DM patients. As for uNKCC2, densitometric analysis (Figure 5(b)) revealed that both DN and NDN patients has a significant higher uNKCC2 excretion compared only to DM patients. However, excretion of both uAQP1 and uNKCC2 was not statistically different between DN and NDN patients.

The post hoc analysis, performed after grouping DN patients according to the class of DN (Figure 4(c)), showed that AQP1 was higher in classes II and III of DN compared with CTR and DM but not with NDN. DN patients in class IV did not show a significant higher uAQP1 excretion compared with the three reference groups. Moreover, the linear regression analysis (Figure 4(d)) showed that uAQP1 did not correlate with DN stage.

As for NKCC2, patients with class II DN showed significantly higher uNKCC2 only compared with DM patients but not with CTR or DN (Figure 5(c)). Linear regression analysis did not show positive or negative relationship between uNKCC2 and the progression of DN (Figure 5(d)).

## 4. Discussion

A number of biomarkers have been exploited in the last decades as tools for early detection of DN [31, 32], yet, to date, none have outperformed microalbuminuria in larger scale, prospective longitudinal studies [33]. However, a number of evidences indicate that albuminuria may not be an optimal marker for the early detection of DN. In fact, about 10–25% of diabetic patients follow the “normoalbuminuria pathway,” showing a progressive decline of GFR without worsening proteinuria [34]. In addition, moderate increases in albumin excretion are associated with a variety of other conditions, including obesity, posture, exercise, diet, smoking, gender, puberty, infection, and inflammation. Therefore, changes in albuminuria may reflect a modification of another disease process that might not be causally related to the development of DN [35]. In the present study, we investigated the possible use of renal aquaporins as biomarkers of tubular damage in patients with histologically proven diagnosis of DN. Recently, studies have shown that not only glomerular damage but also tubulointerstitial damage is an important factor in the progression of DN [36]. Markers of tubular damage could, therefore, be potentially useful in the evaluation of prognosis and for monitoring the effectiveness of treatment in DN. Given the pivotal role of renal AQPs in regulating the fluid balance in the kidney [37], we thought that they might be dysregulated in DN.

The first important finding of this study is that both uAQP5 and uAQP2 were dramatically and selectively upregulated in DN but not in DM and NDN patients. This result likely indicates that increased excretion of uAQP5 and uAQP2 is not a common feature of all forms of chronic kidney disease but rather specific for DN. Both proteins were found to be more abundant in the untreated urine and in the exosome fraction isolated from the urine of DN patients, compared with the three control groups. Two independent experimental approaches were used, an ELISA test on slow-speed urine supernatants and a Western blotting analysis on an ultra-speed sediment, enriched in urine exosomes. Interestingly, the supernatant of the ultra-speed centrifugation did not contain measurable amounts of AQP2 or AQP5 as assessed by ELISA test (data not shown), indicating that both AQP5 and AQP2 in the urine are bound to membrane structures. Regardless of the method used, measurements were normalized for the uCr content. Strikingly, we also demonstrated a strong positive relationship between the abundance of both biomarkers and the progression of DN. Interestingly, the higher sensitivity of the ELISA test, compared to WB, could detect an increase of uAQP5 in DN patients in class II, compared to the three control groups. Patients in class III DN, analyzed by both methods, showed a tendency, although not statistically significant, to a further increase of uAQP5, compared with class II DN. Eventually, patients in class IV showed a dramatic increase of uAQP5. In our study, uAQP2 showed a similar tendency to increase in DN patients in parallel with the progression of the disease. In addition, we confirmed in human kidney biopsies from DN patients that the higher excretion of AQP2 corresponded to a substantial increase of AQP2 at the plasma membrane of principal cells of the collecting ducts. It is well acknowledged that AQP2 enters the apical exosome pathway [27] and the amount of AQP2 excreted into the urine is proportional to that expressed at the apical plasma membrane in a number of physiopathological conditions [38–41].

The concomitant increase of uAQP5 and uAQP2 excretion in DN patients prompted us to investigate the urinary excretion of other membrane proteins expressed in the kidney tubule and involved in the maintenance of the hydroelectrolytes homeostasis. AQP1 and NKCC2 are apically expressed in the proximal tubule and thick ascending limb, respectively [19, 42], and are increased in various types of nephropathy [43, 44]. We showed here that urinary excretion of AQP1 and NKCC2 increased in both DN and NDN patients but this phenomenon was neither positively nor negatively related to the progression of DN. Thus, quantitation of uAQP1 and uNKCC2 would not allow a differential diagnosis between nephropathies and is not useful to classify different stages of DN.

Our results about the upregulation of uAQP5 in DN patients are in line with previous findings from Wu et al. [25] who reported that AQP5 was expressed in the collecting ducts of patients with DN. During the preparation of this manuscript, the same authors demonstrated, with a similar approach, that uAQP5 is upregulated in the urine of DN patients and positively correlated with the stage of DN [45]. In their study, however, they classified DN patients to stages III, IV, and V on the basis of microalbuminuria and macroalbuminuria and increased serum creatinine and limited their analysis to uAQP5 alone. In the present study, we focused our analysis only on patients with histologic-proven diagnosis and staging of DN and analyzed a larger panel of urinary biomarkers. None of the patients enrolled in the present study showed microalbuminuria; thus we could not establish a relationship between AQP5 or AQP2 excretion and this parameter. We also validated the results obtained with the ELISA test on urine samples with a Western blotting analysis on the urine exosome fraction. Our analysis also suggests that, in analogy with AQP2, AQP5 is excreted into the urine through exosomes.

The molecular mechanism responsible for increased expression and excretion of AQP5 and AQP2 in DN patients is unknown and is out of the scope of the present study. However, at least for AQP2, we could hypothesize that AVP might have a key role in this process. Animal studies reported a marked increase of AVP secretion in streptozotocin-induced DM rats and a compensatory increase of AQP2 [46, 47]. Higher AVP levels were also measured in patients with type 2 DM [48] even before they show advanced diabetic complications [49]. From a renal perspective, high AVP levels may be beneficial in early DM by limiting the amount of water required for the excretion of high amount of glucose [46, 50]. In the long term, however, AVP might cause adverse outcomes by aggravating the fluid overload observed in macroalbuminuric DN patients. The adverse effect of AVP on chronic kidney disease (CKD) and DN is well reviewed by Bankir et al. [51]. Most of the work has been done in diabetic animals, which, however, might not be a suitable model to compare with humans. Anyway, the contribution of AVP to DN in humans is supported by several observations: (i) infusion of the AVP analogue desmopressin strongly increases urine albumin excretion in healthy individuals but not in patients with loss-of-function mutations of the type 2 vasopressin receptor [52]; (ii) in epidemiological studies, the percentage of patients with microalbuminuria increased with increasing plasma concentrations of copeptin, a surrogate marker of AVP [53]. Although in the present work we did not measure AVP or copeptin plasma levels in our patients, we can hypothesize that they have sustained levels of circulating AVP which might account for the increased expression/urine excretion of AQP2.

An intriguing hypothesis might be the fact that AVP also regulates AQP5 expression and excretion in the kidney of DN patients. Interestingly, it has been demonstrated that, in human chromosome band 12q13, AQP2 and AQP5 have a closely spaced tandem arrangement [54]. Moreover, in nasal and lung epithelia, cAMP, acting through a PKA/CREB element, upregulated AQP5 expression [55]. Although this mechanism needs to be demonstrated in renal cells, it would nicely fit with the finding presented here.

## 5. Conclusions

Taken together, the results of this pilot investigation demonstrate that urinary excretion of AQP2 and AQP5 might represent a novel urine proteomic signature able to reliably identify diabetic patients with DN using a noninvasive approach. In particular, uAQP5 levels seem to better discriminate DN from NDN patients and may represent a potential novel noninvasive biomarker for the diagnosis and follow-up of DN patients. The ELISA assay showed higher sensitivity, strong specificity, and higher throughput. Besides, it requires a few microliters of unprocessed urine to detect uAQP5. Although our results were obtained on a small cohort of patients, we obtained a strong positive relationship between uAQP5 and the histologic class of DN. A further study, on a larger group of patients, including those with class I DN, would also help to establish how early AQP5 appears in the urine of patients with subclinical signs of DN and to better discriminate between patients in class II and class III of DN.

## Supplementary Material

Supplemental Figure 1: Western blotting analysis of flotillin-1 and Na^+^/K^+^-ATPase expression in urinary exosomes.
Supplemental Figure 2: Correlation analysis between uAQP5 and uACR or CKD-EPI.
Supplemental Figure 3: Correlation analysis between uAQP2 and uACR or CKD-EPI.

## Figures and Tables

**Table 1:** Demographic and clinical characteristics of 12 diabetic patients (DM), 11 diabetic patients with nondiabetic nephropathy (NDN), and 12 patients with diabetic nephropathy (DN). Data are expressed as the mean ± standard error of the mean (SEM). Statistical analysis was performed using one-way ANOVA followed by Newman-Keuls multiple comparison test. *P* values < 0.05 were considered statistically significant.

	Diabetes mellitus (DM)	Nondiabetic nephropathy (NDN)	Diabetic nephropathy (DN)	*P* value	
Number of patients	12	11	12		

Sex (female/male)	2/10	4/7	2/10		

Age (years)	56 ± 4	58 ± 5	63 ± 4	DM versus NDN	*P* = 0.88
				DM versus DN	*P* = 0.25
				NDN versus DN	*P* = 0.38

uCr (mg/dL)	188 ± 20	81 ± 14	89 ± 22	DM versus NDN	*P* = 0.0003
				DM versus DN	*P* = 0.0028
				NDN versus DN	*P* = 0.75

CKD EPI	99 ± 5	64 ± 13	42 ± 9	DM versus NDN	*P* = 0.016
				DM versus DN	*P* < 0.0001
				NDN versus DN	*P* = 0.17

uACR (mg/g)	23 ± 14	1156 ± 402	2580 ± 677	DM versus NDN	*P* = 0.0076
				DM versus DN	*P* = 0.001
				NDN versus DN	*P* = 0.092

MAP (mmHg)	92 ± 4	95 ± 4	100 ± 4	DM versus NDN	*P* = 0.61
				DM versus DN	*P* = 0.12
				NDN versus DN	*P* = 0.27

Plasma TG (mg/dL)	117 ± 22	216 ± 31	253 ± 41	DM versus NDN	*P* = 0.015
				DM versus DN	*P* = 0.0076
				NDN versus DN	*P* = 0.49

Total CHO (mg/dL)	157 ± 10	164 ± 12	165 ± 11	DM versus NDN	*P* = 0.64
				DM versus DN	*P* = 0.61
				NDN versus DN	*P* = 0.98

BMI (kg/m^2^)	29 ± 2	27 ± 1	30 ± 1	DM versus NDN	*P* = 0.37
				DM versus DN	*P* = 0.69
				NDN versus DN	*P* = 0.10

Antidiabetic agents	Insulin = 6 ADO = 6	Insulin = 3 ADO = 8	Insulin = 5 ADO = 6 None = 1		

uCr, urine creatinine; CKD-EPI (Chronic Kidney Disease Epidemiology Collaboration), estimated glomerular filtration rate; uACR, urine albumin/urine creatinine; MAP, mean arterial pressure; Plasma TG, plasma triglyceride levels; Total CHO, total plasma cholesterol levels; BMI, body mass index.

**Table:** (a) Patients with diabetic nephropathy (DN)

Patient	K-DOQI stage (CKD-EPI)	Histologic class of DN	Interstitial fibrosis and tubular atrophy	Interstitial inflammation	Arteriolar hyalinosis	Large vessels arteriosclerosis
1	4	IV	3	1	2	1
2	5	IV	3	2	2	1
3	3	IIa	2	1	1	2
4	5	IV	3	1	1	1
5	3	III	2	1	2	1
6	1	IIa	2	1	2	1
7	3	III	2	1	2	1
8	4	III	2	1	1	1
9	3	III	3	2	2	1
10	3	IIb	2	1	2	1
11	1	IIb	1	1	1	1
12	3	III	3	1	1	1

**Table:** (b) Patients with nondiabetic nephropathy (NDN)

Patient	K-DOQI stage (CKD-EPI)	Histologic diagnosis	Interstitial fibrosis and tubular atrophy	Interstitial inflammation	Arteriolar hyalinosis	Large vessels arteriosclerosis
1	1	Nephroangiosclerosis	2	0	1	1
2	3	FSGS (NOS)	1	0	1	0
3	1	Lupus GN (V)	2	0	0	0
4	5	Interstitial GN	3	1	1	1
5	5	Endocapillary and extracapillary GN	2	1	1	1
6	4	Nephroangiosclerosis	2	2	2	1
7	1	Membranous GN II	1	0	2	1
8	1	FSGS (perihilar)	2	1	1	1
9	2	Membranous GN I	2	0	1	1
10	2	Membranous GN II	1	0	1	1
11	3	FSGS (NOS)	2	2	2	1
