# Supplementary material for: Urinary Excretion of Kidney Aquaporins as Possible Diagnostic Biomarker of Diabetic Nephropathy
Source: J Diabetes Res. 2017 Jan 26;2017:4360357. doi: 10.1155/2017/4360357 (PMC5299189; doi:10.1155/2017/4360357)
Supplement: Supplementary file 1 — Supplemental Figure 1: Western blotting analysis of flotillin-1 and Na+/K+-ATPase expression in urinary exosomes. Supplemental Figure 2: Correlation analysis between uAQP5 and uACR or CKD-EPI. Supplemental Figure 3: Correlation analysis between uAQP2 and uACR or CKD-EPI. [file 4360357.f1.zip › Supplemental material/LEGENDS TO SUPPLEMENTAL FIGURES.docx]

LEGENDS TO SUPPLEMENTAL FIGURES

**Supplemental Figure 1. Western blotting analysis of flotillin-1 and Na+/K+-ATPase expression in urinary exosomes**.

Exosomes were isolated from urine of DM, NDN and DN patients by differential ultracentrifugation (see materials and methods), separated by SDS-PAGE and analyzed by Western blotting with antibodies against flotillin-1 and Na+/K+-ATPase. An equal volume (10 μl) of exosome was loaded in each lane. 3T3-L1 was used as positive control. A strong signal for flotillin-1 was clearly detected and reported while the presence of protein for Na+/K+-ATPase was not detected in all exosome samples. Here are reported the representative results obtained in three patients per group. Similar results were obtained in the entire group of patients. Experiments were repeated three times with comparable results.

**Supplemental Figure 2. Correlation analysis between uAQP5 and uACR or CKD-EPI.**

Spearman correlation test was performed to calculate the correlation between uAQP5 (x axis) and uACR or CKD-EPI (y axis). Each point in the plot represented a patient. (**A, B**) In DM patients uAQP5 positively and significantly correlated with uACR (r=0.64, p=0.02) but it did not correlate with CKD-EPI (r=-0.4, p=0.19). (**C, D**) In NDN patients uAQP5 correlate neither with uACR (r=0.59, p=0.05) nor with CKD-EPI (r=0.08, p=0.80). (**E, F**) In DN patients uAQP5 of did not correlate with uACR (r=0.35, p=0.26) but it negatively and significantly correlated with CKD-EPI (r=-0.61, p=0.034). r, Spearman correlation coefficient.

**Supplemental Figure 3. Correlation analysis between uAQP2 and uACR or CKD-EPI.**

Spearman correlation test was performed to calculate the correlation between uAQP2 (x axis) and uACR or CKD-EPI (y axis). Each point in the plot represented a patient. (**A, B**) In DM patients, uAQP2 correlated neither with uACR (r=0.38, p=0.21) nor with CKD-EPI (r=-0.44, p=0.14). (**C, D**) NDN patients did not show a statistically significant correlation between uAQP2 and uACR (r=-0.33, p=0.31) or CKD-EPI (r=0.22, p=0.50). (**E, F**) In DN patients, uAQP2 did not correlate with uACR (r=0.27, p=0.39) but it negatively and significantly correlated with CKD-EPI (r=-0.58, p=0.04). r, Spearman correlation coefficient.
